# Venom biotechnology: casting light on nature’s deadliest weapons using synthetic biology

**DOI:** 10.3389/fbioe.2023.1166601

**Published:** 2023-05-03

**Authors:** Tim Lüddecke, Anne Paas, Richard J. Harris, Lea Talmann, Kim N. Kirchhoff, André Billion, Kornelia Hardes, Antje Steinbrink, Doreen Gerlach, Bryan G. Fry, Andreas Vilcinskas

**Affiliations:** ^1^ Department of Bioresources, Fraunhofer Institute for Molecular Biology and Applied Ecology, Giessen, Germany; ^2^ LOEWE Centre for Translational Biodiversity Genomics (LOEWE-TBG), Frankfurt am Main, Germany; ^3^ Venom Evolution Lab, School of Biological Sciences, The University of Queensland, Brisbane, QLD, Australia; ^4^ Institute for Molecular Biosciences (IMB), The University of Queensland, Brisbane, QLD, Australia; ^5^ Syngenta Crop Protection, Stein, Switzerland; ^6^ BMBF Junior Research Group in Infection Research “ASCRIBE”, Giessen, Germany; ^7^ Institute for Insect Biotechnology, Justus Liebig University of Giessen, Giessen, Germany

**Keywords:** synthetic biology, biodiscovery, functional genomics, organoids, CRISPR, RNAi, heterologous expression, biosensors

## Abstract

Venoms are complex chemical arsenals that have evolved independently many times in the animal kingdom. Venoms have attracted the interest of researchers because they are an important innovation that has contributed greatly to the evolutionary success of many animals, and their medical relevance offers significant potential for drug discovery. During the last decade, venom research has been revolutionized by the application of systems biology, giving rise to a novel field known as venomics. More recently, biotechnology has also made an increasing impact in this field. Its methods provide the means to disentangle and study venom systems across all levels of biological organization and, given their tremendous impact on the life sciences, these pivotal tools greatly facilitate the coherent understanding of venom system organization, development, biochemistry, and therapeutic activity. Even so, we lack a comprehensive overview of major advances achieved by applying biotechnology to venom systems. This review therefore considers the methods, insights, and potential future developments of biotechnological applications in the field of venom research. We follow the levels of biological organization and structure, starting with the methods used to study the genomic blueprint and genetic machinery of venoms, followed gene products and their functional phenotypes. We argue that biotechnology can answer some of the most urgent questions in venom research, particularly when multiple approaches are combined together, and with other venomics technologies.

## 1 Introduction

The ability to impose “chemical warfare” in the form of venom is widespread throughout the animal kingdom (Schendel et al., 2019). Venom is a functional trait that has been optimized by evolution to fulfil the three core functions of predation, defense and competitor deterrence, along with side functions such as immunomodulation and sexual communication (Schendel et al., 2019). Venoms are complex and diverse mixtures of powerful bioactive molecules (Fry et al., 2009; Casewell et al., 2013; Schendel et al., 2019). The astonishing molecular diversity, bioactivity and medical significance of venoms promoted early scientific interest that continues to this day (Kasturiratne et al., 2008; Casewell et al., 2013; Robinson et al., 2017; Herzig et al., 2020; Zancolli and Casewell, 2020).

During its maturation as a scientific discipline, venom research has passed through several stages of development marked by certain core technologies that were replaced or supplemented as new technologies emerged. For example, during the infancy of zootoxinology in the late 1800s, it was common to study crude sample materials and isolate single components. This was mostly achieved by applying laborious manual extraction protocols that were ultimately replaced by preparative chromatography, as previously discussed in the context of amphibian toxins (Lüddecke et al., 2018). The efficiency of venom fractionation increased following the development of advanced mass spectrometry, structural biology and *in vitro* bioassay platforms, which allowed the composition and biochemistry of several venom systems to be analyzed in detail. The most recent era of venom research was sparked by the exponential growth of bioinformatics, next-generation sequencing and proteomics (Calvete and Lomonte, 2015; Dutertre et al., 2015; Calvete, 2017; Drukewitz and von Reumont, 2019). The term venomics was chosen to describe the application of these new technologies to venom systems, rapidly accelerating venom research and overcoming many of the earlier hurdles (Escoubas, 2006; Calvete et al., 2007; Calvete, 2017; von Reumont et al., 2022). For example, thanks to the high sensitivity of modern methods, venom components can now be analyzed using limited amounts of sample material. This has allowed the inclusion of species that were formerly either too rare for the collection of meaningful amounts of venom, or too small to deliver sufficient venom yields (de Graaf et al., 2009; Bouzid et al., 2014; Lewis Ames et al., 2016; Walker et al., 2018; Walker et al., 2021; Casewell et al., 2019; Krämer et al., 2019; Schmidt et al., 2019; Lüddecke et al., 2020; Lüddecke et al., 2022; Hurka et al., 2022; von Reumont et al., 2022).

At the time of writing, venom research is experiencing yet another major advance based on the use of synthetic biology for the analysis of venom function and evolution. Synthetic biology is an important branch of biotechnology involving the design or redesign of biological systems, promoting new advances in biomedicine, pharmacology and industrial production (El Karoui et al., 2019). The application of synthetic biology to venom research can therefore achieve great advances in the understanding and utilization of the deadliest weapons found in the animal kingdom, as highlighted by some of the important recent discoveries discussed in this article. We therefore expect synthetic biology to become transformative in future venom research as it has been for other disciplines.

**FIGURE 1:**
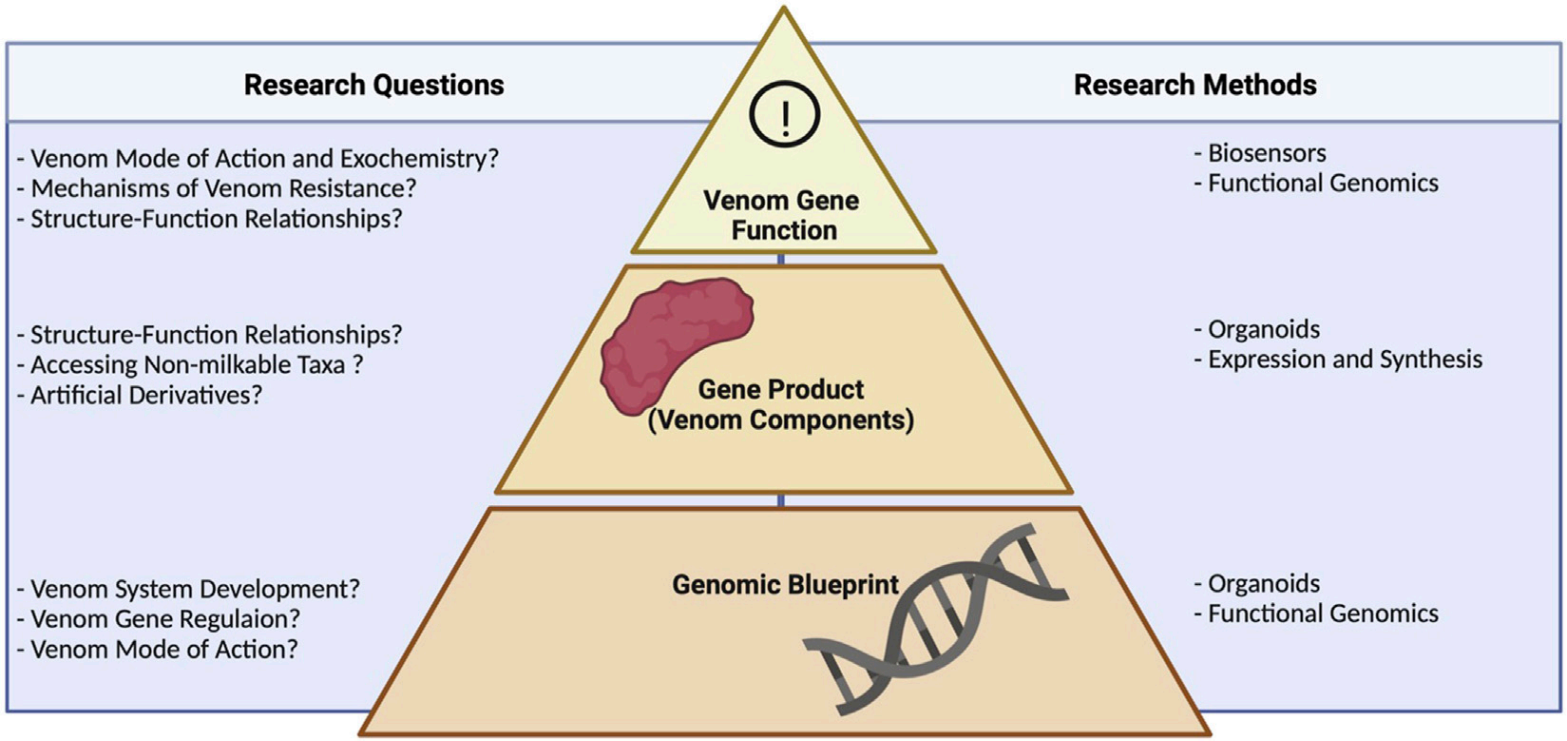
Research questions in venom biotechnology and the methods used to address them, showing the relevance of different levels of biological organization. Technologies, such as organoids and functional genomics, working at the genomic level, help to unravel venom system development, gene regulation, and modes of action. Approaches targeting the gene product include heterologous expression and, again, organoids. These guide studies on structure–function relationships, allow access the venom of non-milkable organisms, and facilitate the production of artificial derivatives of known venom components. Biosensors and functional genomics enable the interrogation of venom systems on the functional level, addressing questions on mode of action, resistance mechanisms, and structure–function relationships. Note that several technologies help to answer biological questions about venom systems across different levels of biological organization.

Despite the impact of synthetic biology in modern venom research, we nevertheless lack a comprehensive analysis of the cutting-edge technologies and their application to venom systems. Here we aim to fill this important gap by summarizing the most important synthetic biology technologies applied in venom research, as well as their implications and current limitations. We discuss how these technologies are currently deployed in venom research and anticipate key developments. We argue in particular that the combinatorial use of such technologies, especially in tandem with venomics, will allow us to discover the diverse mechanisms underlying venom production and activity, leading to novel applications that benefit humanity. This evaluation of venom biotechnology will follow the natural hierarchy of biological information, starting from the gene and progressing via the product to gene function (Figure 1). Therefore, we start by outlining how biotechnology has been used at the level of genomes to study the genetic basis of venom and venom systems. This is followed by a discussion of the methods used to gain access to the gene products (toxins) and finally how they have been used to develop systems that allow the experimental analysis of toxin functions.

## 2 Manipulating genetic information: Studying venom systems by functional genomics

**FIGURE 2:**
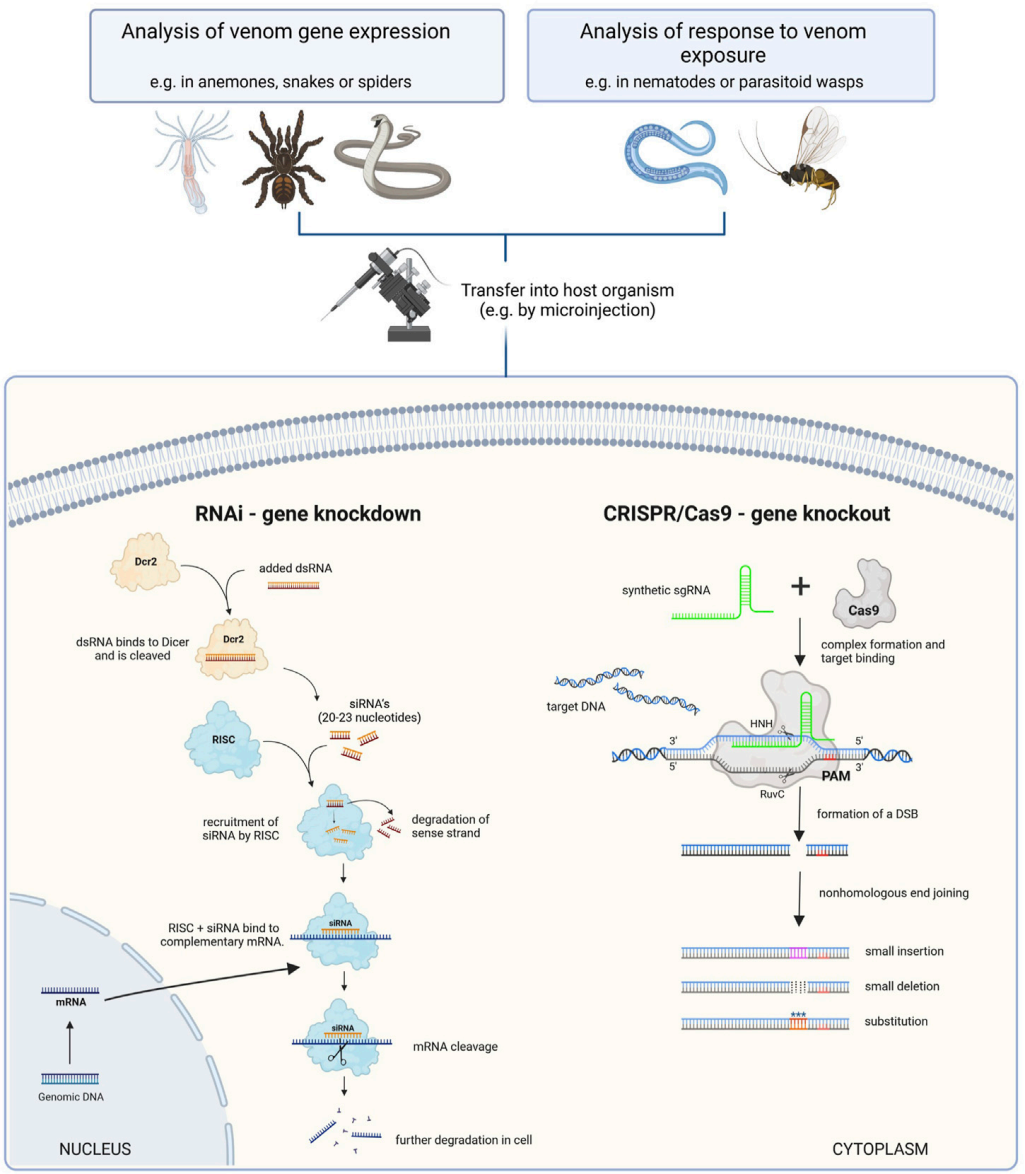
Comparison of functional genomics tools that enable loss-of-function studies in venom research. Left: RNAi is used to induce the temporary knockdown of targeted genes by degrading mRNAs. At first double-stranded RNA (dsRNA) is injected and binds to the Dicer protein, where it is cleaved into small interfering RNAs (siRNA). These are subsequentially recruited to the RNA-induced silencing complex (RISC), which binds to complementary mRNA and facilitates its degradation. Thus, the corresponding gene is temporarily not translated. Right: CRISPR/Cas9 is typically used to introduce double-strand breaks that are erroneously repaired, leading to a stable knockout mutation. Here, single guide RNA (sgRNA) is injected and binds to the Cas9 protein. The resulting complex interacts with target DNA complementary to the sgRNA. Cas9 cleaves the DNA upstream to the protospacer adjacent motif (PAM) and leads to nonhomologous end joining, allowing to precisely add substitutions, smaller insertions and deletions to the sequence in dependence of experimental conditions. Deletions lead to permanent removal of targeted sequences and thus the corresponding gene can not be expressed. The figure is adapted from (Knorr et al., 2013; Jiang and Doudna, 2017; Bharathkumar et al., 2021).

Some long-lasting mysteries in venom research relate to genetic interactions in the context of venom production, regulation, system development, maintenance, and the biochemical interactions of venom components. These can be addressed by functional genomics, in which the genetic basis of venom phenotypes is tested by introducing DNA modifications or manipulating gene expression at the RNA level. In particular, when applied to cell cultures or venomous laboratory organisms, functional genomics can identify the genetic machinery underlying venom systems, provide insights into the pathobiochemistry of venoms, and lead to the discovery of new treatments (Section 2.2). Functional genomics tools and methods used in venom research thus far are mostly loss-of-function methods, which silence genes or knock them out completely. These approaches are compared in Figure 2.

### 2.1 Gene silencing by RNA interference

RNA interference (RNAi) is a functional genomics method typically involving the introduction of double stranded RNA (dsRNA) matching an endogenous gene sequence, which results in the potent and specific, yet transient, silencing of that gene by triggering the degradation of the corresponding mRNA (Setten et al., 2019). It is based on an ancient defense mechanism that evolved to silence the genes of invading pathogens. More recently, it has been used to disentangle the basis of venom systems by selectively inactivating specific components (Setten et al., 2019).

RNAi was pioneered in the nematode *Caenorhabditis elegans*, and the first use of RNAi for venom research was also in that species, focusing on latrophilin-dependent resistance against black widow (*Latrodectus* spp.) venom (Mee et al., 2004). However, RNAi has mainly been used to study the effects of certain venom components during envenomation, particularly in parasitoid wasps and their insect hosts. Rather than killing their prey, parasitoid wasps use their venom to paralyze insect hosts and modulate their metabolism before laying eggs. Accordingly, parasitoids can be used to study the functional aspects of envenomation by knocking down genes encoding venom components (Colinet et al., 2014). RNAi was instrumental in the analysis of venom function during wasp life cycles and helped to deduce the evolutionary history of glycoside hydrolase chitinases (Martinson et al., 2016). Knockdown experiments in *Nasonia vitripennis* revealed that chitinases primarily trigger immune responses that defend the host against fungi (Martinson et al., 2016). In a similar approach, knockdown experiments targeting the *N. vitripennis* venom component venom Y suggested that its role is the detoxification and mitigation of other venom components, in addition to regulating the host immune system (Martinson et al., 2019). The knockdown of α-amylase in *Pteromalus puparum* venom negatively affected larval development, suggesting that amylases regulate host metabolism and developmental processes (Wang et al., 2020a).

### 2.2 CRISPR/Cas9 mutagenesis

CRISPR/Cas9 is a genome editing technology that is mainly used to knock out specific genes by targeted mutagenesis. In the typical approach, the endonuclease Cas9 is targeted to a particular site in the genome by a guide RNA (gRNA). Cas9 then introduces a precisely-targeted double-strand break, which is repaired by the erroneous non-homologous end joining (NHEJ) pathway resulting in small insertions or deletions (Jiang and Doudna, 2017). When combined with cell culture and genome sequencing, CRISPR/Cas9 technology can generate loss-of-function mutants in selected cell lines, thus allowing the rapid analysis of emerging phenotypes. More sophisticated applications include the introduction of precise deletions, insertions or nucleotide replacements by providing a donor template to stimulate homology-dependent repair, and the use of modified versions of the Cas9 nuclease lacking functional endonuclease domains to regulate gene expression (Ford et al., 2019; Gupta et al., 2019; Manghwar et al., 2019; Hendriks et al., 2020). Whereas the effects of RNAi are transient, CRISPR/Cas9 knockout causes a permanent loss of function.

Genome-wide CRISPR/Cas9 knockout (GeCKO) screening has recently been used to dissect venom pathobiochemistry. In a pioneering study, this approach was used to unravel the mechanisms underlying the cytotoxicity of box jellyfish (*Chironex fleckeri*) venom (Lau et al., 2019). The authors combined CRISPR/Cas9 mutagenesis and pharmacological inhibition to show that box jellyfish cytotoxicity targets the necroptotic and apoptotic machinery of its victims. Based on these initial findings, the mutation of 19,050 genes by GeCKO following treatment with *C. fleckeri* venom revealed a number of genes relevant for venom resistance, including the ATPase plasma membrane Ca^2+^ transporting gene *ATP2B1*. The analysis of biological pathways enriched in the surviving human near-haploid cells (HAP1) suggested a pivotal role for cholesterol and sphingolipids during the envenomation process, which was confirmed experimentally. The authors also found that substances used to remove cholesterol from cell membranes (such as methyl-β-cyclodextrins and 2-hydroxypropyl-β-cyclodextrins) confer concentration-dependent resistance against box jellyfish venom. Interestingly, the protective action of these compounds was apparent even when administered 15 min post-envenomation, which suggests a significant therapeutic potential (Lau et al., 2019). As well as identifying a potential antidote for a difficult-to-treat envenomation, this study also enabled the global molecular dissection of envenomation pathways from a victim’s perspective. Most studies of venom systems and their mode of action involve traditional assays, which limit the analysis to a subset of venom targets. In contrast, CRISPR/Cas9 technology reveals a holistic perspective of venom activity and targets. Such screening experiments are likely to become more common because they offer a powerful way to pinpoint the mode of action of crude venom and individual toxins, which are largely unknown within the animal kingdom.

The use of CRISPR/Cas9 in venom research is not restricted to pathobiochemical and mechanistic questions but can also address the development and genetic architecture of venom systems. In the anemone *Nematostella vectensis*, CRISPR/Cas9 was used to study the development of cnidocytes, the venom delivery system of cnidarians and the first venom system that evolved in metazoans (Sunagar et al., 2018). In this case, CRISPR/Cas9 was not used to knock out target genes but instead as a knock-in system to generate transgenic *N. vectensis* lines expressing fluorescent reporter proteins in cnidocytes. Tentacle dissection and subsequent fluorescence-activated cell sorting (FACS) allowed the generation and transcriptomic analysis of stable cnidocyte cell lines throughout the maturation process, leading to the identification of a gene set that may be involved in cnidocyte formation (Sunagar et al., 2018). The role of these genes was confirmed by subsequent knockdown, which disrupted the process. Although this work did not focus on the *N. vectensis* venom system, it nevertheless highlights the potential of CRISPR/Cas9 technology to shed light on the morphological origin of venom systems and the underlying genetic machinery. CRISPR/Cas9 has been used thus far to study cnidarian systems but will be expanded in the future to other venomous species. For example, a new subset of CRISPR/Cas9 technologies enabling the establishment of mutant lines of the parasitoid wasp *N. vitripennis* is likely to provide insights into the venom system of this species in the near future (Li et al., 2017).

### 2.3 Challenges and promises of functional genomics in venom research

Functional genomics is a powerful tool for the analysis of venom systems but there are two major prerequisites. The first is the availability of genome-scale data to facilitate the design of efficient probes against genes of interest. The limited availability of such genomes has hindered venom research in the past, but a broad range of genomes representing different venomous lineages has been sequenced more recently, including lesser-known species such as the solenodon (*Solenodon paradoxus*) and enigmatic species as the Komodo dragon (*Varanus komodoensis*), Indian cobra (*Naja naja*), king cobra (*Ophiophagus hannah*), tiger rattlesnake (*Crotalus tigris*), and wasp spider (*Argiope bruennichi*) (Vonk et al., 2013; Casewell et al., 2019; Lind et al., 2019; Suryamohan et al., 2020; Margres et al., 2021; Sheffer et al., 2021).

The second is the availability of venom-secreting cell lines or venomous animal lineages that can be manipulated using such techniques. A taxonomically diverse range of cell lines has been established, but cultures from venom glands are still rare. Even so, some attempts have been made to maintain venom glands from snakes and insects in culture, which culminated in the recent development of whole venom gland organoids as discussed in Section 3 (Hink and Butz, 1985; Duarte et al., 1999; Carneiro et al., 2006; Post et al., 2020). Furthermore, laboratory strains and functional genomics protocols are already available for some venomous animal lineages, such as anemones, spiders, and wasps, and these may be expanded to include more venom systems in the future (Prpic et al., 2008; Colinet et al., 2014; Feitosa et al., 2017; Sunagar et al., 2018). Of particular potential in this context are spiders, especially the common house spider *Parasteatoda tepidariorum*. This species is a close relative of the black widow (genus *Latrodectus*) and an already established model organism for developmental and evolutionary-developmental questions (Prpic et al., 2008; Hilbrant et al., 2012; Oda and Akiyam-Oda., 2020). A variety of RNAi-based studies have been performed on this species and its exploitation to address future venom related questions appears as a logical next step.

Another potentially important development is heralded by the growing body of evidence suggesting a role of microRNAs (miRNA) within venom systems. Functionally related to the process of RNAinterference, miRNAs are involved in post-transcriptional gene silencing and thus are involved in gene regulatory processes (He et al., 2004). They are known to be major players of gene regulation across the animal kingdom and are heavily investigated for the development of translational tools, e.g., as weapons against pest insects (He et al., 2004; Bally et al., 2020). Interestingly, recent studies showed that miRNAs are also involved in the regulation of venom genes and are widely expressed in venom glands (Durban et al., 2013; Vonk et al., 2013; Durban et al., 2017). They also are induced in tissue after exposure to venom toxins (Nassimento et al., 2022). Although the role and diversity of miRNAs has been only seldomly studied, the available literature suggests that they are of hitherto unrecognized importance. Future studies should therefore aim to investigate miRNA profiles of venom systems from different species and establish their role. This could lead to the development of future miRNA-based tools for functional genomics and shed new light on the biological aspects of venom gene regulation.

In light of the growing body of available genomes, cell cultures, animal lineages suitable for functional genomics and the emerging role of miRNAs, it is likely that such technologies will gain momentum in the future and that functional genomics will continue to provide key mechanistic insights into unexplored venom systems.

## 3 How synthetic biology enables access to venom gene products

The level of biological organization above the genome is the gene product, which in the context of venom systems generally refers to peptide and protein toxins (Fry et al., 2009). The traditional approach to access such components is the collection of crude venom followed by chromatography to generate fractions. However, this is only possible in a limited range of taxa due to the small size and venom yield of many venomous organisms, which has excluded such species from analysis in the past (Section 3.2) (Von Reumont, 2018; Herzig et al., 2019; Lüddecke et al., 2019). Venom biotechnology is now driving the functional analysis of toxins because it provides a methodological framework to circumvent these limitations and access scarce toxins via other routes. Approaches include the development of artificial venom glands or the use of sequence information combined with the production of synthetic or recombinant proteins and peptides.

### 3.1 Venom gland organoids for the analysis of venom systems

Stable cell culture systems are necessary for several aspects of venom system biology, particularly those involving functional genomics, but only a few primary cell cultures have been established thus far (Sells et al., 1989; Duarte et al., 1999; Carneiro et al., 2006; Yamanouye et al., 2007). This has been addressed recently by the development of snake venom gland organoids, which are stem cell-derived self-organizing 3D structures that resemble the parent organ (Clevers, 2016). Importantly, such mini-organs display many of the natural features of the tissues they mimic (Clevers, 2016). In a pioneering study, venom gland organoids were derived from nine venomous snakes: five elapids and four viperids (Post et al., 2020). This study was remarkable because it reported an easy, reproducible way to produce organoids from snake venom glands and keep them stable in culture for multiple passages (Post et al., 2020; Puschhof et al., 2021). RNA-sequencing (RNA-Seq) experiments on cape cobra (*Naja nivea*) and cape coral snake (*Aspidelaps lubricus cowlesi*) cultures revealed that the organoids strongly express toxin-related transcripts and a profile of venom factors similar to natural venom glands (Post et al., 2020). In addition to the transcriptomic assessment of venom expression, the authors used liquid chromatography coupled with mass spectrometry (LC/MS) to compare protein extracts from *A. l. cowlesi* organoids and crude venom. The organoids were also shown to express venom proteins and peptides, which was supported by further bioactivity studies in which organoid extracts were used in OrganoPlate organ-on-a-chip assays, and by the generation of a fluorescent reporter organoid line using CRISPR/Cas9 enabling the detection of three-finger toxin (3FTx) proteins (Trietsch et al., 2017; Post et al., 2020).

**FIGURE 3:**
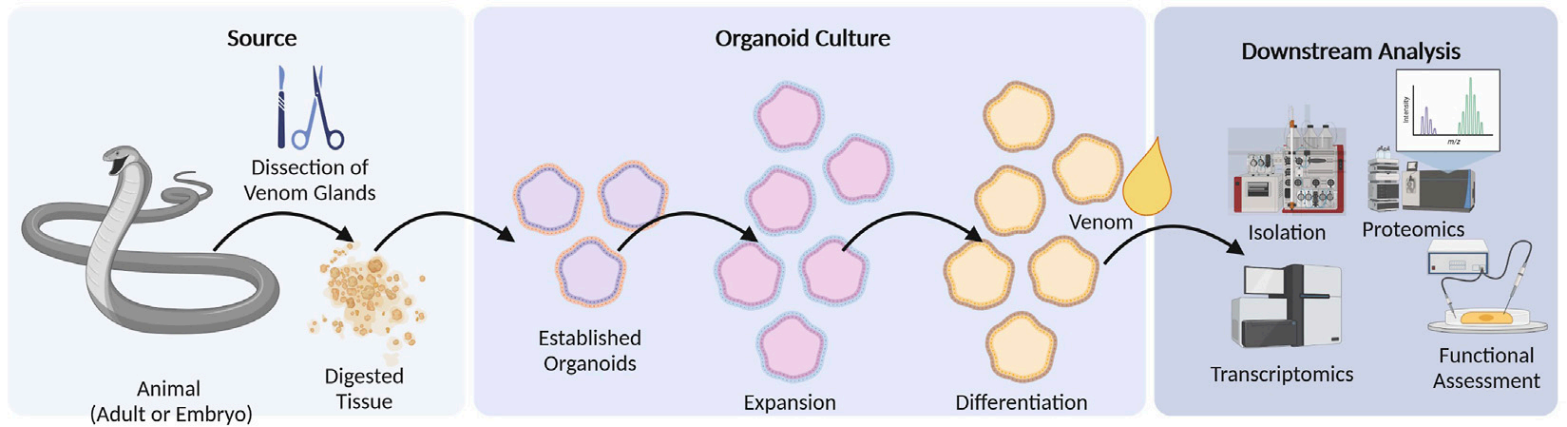
Organoid culture workflow. Venom glands are obtained by dissection from adult or embryonic specimens of the target species. Following tissue digestion, they are established in cell culture where they undergo expansion and finally differentiation. Differentiated organoids resemble artificial venom glands in a controllable cell culture system and may be used to obtain crude venom for downstream venomics analysis or for the isolation of specific toxins. They may also be used as model systems to study the genetic and developmental basis of venom glands.

The establishment of organoids for venom systems (Figure 3) provides a versatile research tool that allows the genetic and cellular analysis of venom systems, focusing on gene regulation, protein trafficking and posttranslational modifications (Post et al., 2020; Zancolli and Casewell, 2020; Puschhof et al., 2021). In contrast to other systems, organoids are easy to cultivate in the laboratory, allowing the experimental manipulation of a large number of replicates (Zancolli and Casewell, 2020; Puschhof et al., 2021). A major obstacle for organoid systems can be the limited time they can be maintained in a stable manner (Hofer and Lutolf, 2021). In case of the established snake venom gland organoids, the utilization of a mammalian growth factor cocktail seemingly allows a virtual indefinite culturing (Post et al., 2020). The compartmentalized nature of venom systems, in which distinct areas produce different components of the venom, are also maintained in venom gland organoids (Tayo et al., 2010; Hu et al., 2011; Undheim et al., 2014; Valente et al., 2018; Post et al., 2020; Schmidtberg et al., 2021). Furthermore, given that organoids faithfully produce venom components, they can be used to culture venom glands from animals that are difficult to study even by venomics, to obtain larger amounts of venom for functional analysis. Moreover, the implementation of stable organoids may circumvent the need to keep multiple animals in a lab and may cause a reduction of experimental animals in future venom studies.

### 3.2 Do it yourself: the production of venom components

#### 3.2.1 Chemical synthesis

Traditional fractionation approaches are unsuitable for venoms that are difficult to access in sufficient quantities, so other methods are required. Total chemical synthesis can be used to produce short venom peptides with few disulfide bridges. This method was recently used to produce challenging inhibitor cysteine knot (ICK) peptides from scorpions, assassin flies and anemones (Pennington et al., 1995; Park et al., 2012; Drukewitz et al., 2018; Juichi et al., 2018), and is a straightforward way to produce simple antimicrobial peptides (AMPs) from sequence data (Torres-Larios et al., 2000; Zelezetsky et al., 2005; Rifflet et al., 2012). For example, venom AMPs from urodacid scorpions and pseudoscorpions were produced by solid-phase synthesis for testing against pathogens (Luna-Ramirez et al., 2017a; Krämer et al., 2022). Likewise, short peptide toxins from myrmicine ants were synthesized and tested for their insecticidal activities (Heep et al., 2019a; Heep et al., 2019b). A peptide identified in fire salamander (*Salamandra salamandra*) skin poison was also synthesized and tested for toxicity and antioxidant activity (Plácido et al., 2020).

#### 3.2.2 The heterologous expression of toxins in prokaryote cells

Chemical synthesis is unsuitable for peptides more than 50 amino acids in length, so longer peptides and proteins, as well as those with more complex disulfide bonds, must be produced using recombinant DNA technology. The bacterium *Escherichia coli* is the most widely used production host for venom peptides (Saikia et al., 2021), and strains carrying the phage T7 RNA polymerase gene under the control of the *lac*UV5 promoter can be used for inducible expression with the lactose analog isopropyl *ß*-d-1-thiogalactopyranoside (IPTG), thus boosting expression levels independently of cell growth (Marschall et al., 2017; Terol et al., 2021). For the production of toxins, leaky expression can inhibit cell growth and thus limit the cell density in culture. Some strains, such as BL21 (DE3) pLysS, carry an additional plasmid encoding T7 lysozyme, a natural inhibitor of T7 RNA polymerase, which suppresses basal expression prior to induction (Studier, 1991). Additionally, pLysS increases the stability of plasmids carrying toxin genes (Minea et al., 2005). IPTG is a potent inducer even at very low concentrations, so tunable expression has required the development of strains like Tuner (Novagen), which has been used to produce a venom protein from the southern copperhead snake (Minea et al., 2005).

One disadvantage of *E. coli* is that it cannot produce complex toxins with multiple disulfide bonds in the cytosol because disulfide bonds are quickly reduced by reductases and small-molecule reductants (Berkmen, 2012). Therefore, the toxins accumulate as insoluble inclusion bodies. This can be addressed by using redox-engineered *E. coli* strains such as Origami (Novagen), with an oxidizing cytosol (Minea et al., 2005; Clement et al., 2016). The co-expression of a disulfide isomerase such as DsbC in redox-engineered *E. coli* cells further enhances the oxidative folding of proteins (Lobstein et al., 2012). Sermadiras et al. (2013) used the *E. coli* strain SHuffle T7 Express lysY for the production of a neurotoxin peptide from the Chinese bird spider (*Ornithoctonus huwena*) and maximized the production of the soluble and correctly-folded peptide by lowering the cultivation temperature to 16°C overnight.

Recombinant proteins with disulfide bonds can be expressed in the cytoplasm of *E. coli* cells with the reducing pathways intact by applying the CyDisCo system, thanks to the pre-expression or co-expression of a sulfhydryl oxidase and a disulfide isomerase (Nguyen et al., 2011; Gąciarz et al., 2017). Nielson et al. (2019) used the CyDisCo system to produce a conotoxin from a venomous marine cone snail in the cytosol of *E. coli*. Bertelsen et al. (2021) combined the pLemo tunable expression system with two CyDisCo variants to enhance the soluble expression of a conotoxin from *Conus striatus*. Applying the pcsDisCo Tune system increased the amount of conotoxin peptide in the soluble fraction by up to 4.1-fold.

The expression of disulfide-rich proteins can also be achieved by targeting the protein to the periplasm, although this accounts for only 8%–16% of the cell volume (Joachim et al., 2019). Nevertheless, a periplasmic expression system was designed to produce the challenging ICK peptides that dominate invertebrate venoms (Klint et al., 2013). This system has been used to express ICK toxins from centipedes, spiders and cone snails (Klint et al., 2013; Loening et al., 2013) as well as funnel web spider (*Hadronyche*) neurotoxins with a double ICK motif (Chassagnon et al., 2017).

Recombinant venom components are usually expressed as fusion proteins with thioredoxin (TrxA), maltose-binding protein (MBP) or disulfide isomerase (DsbC) domains to improve solubility and promote the formation of disulfide bonds, and/or with tags such as His_6_ to facilitate purification. The tags must be cleaved after expression, which may leave residual amino acids attached to the toxin. In contrast, the cleavage sites of Factor Xa and enterokinase allow the complete removal of the fusion partner, although some nonspecific cleavage may occur. An important advantage of the fusion strategy is that the toxin remains inactive until the fusion partner is removed, thus protecting the production host and also the researcher from the toxin’s effects.

**FIGURE 4:**
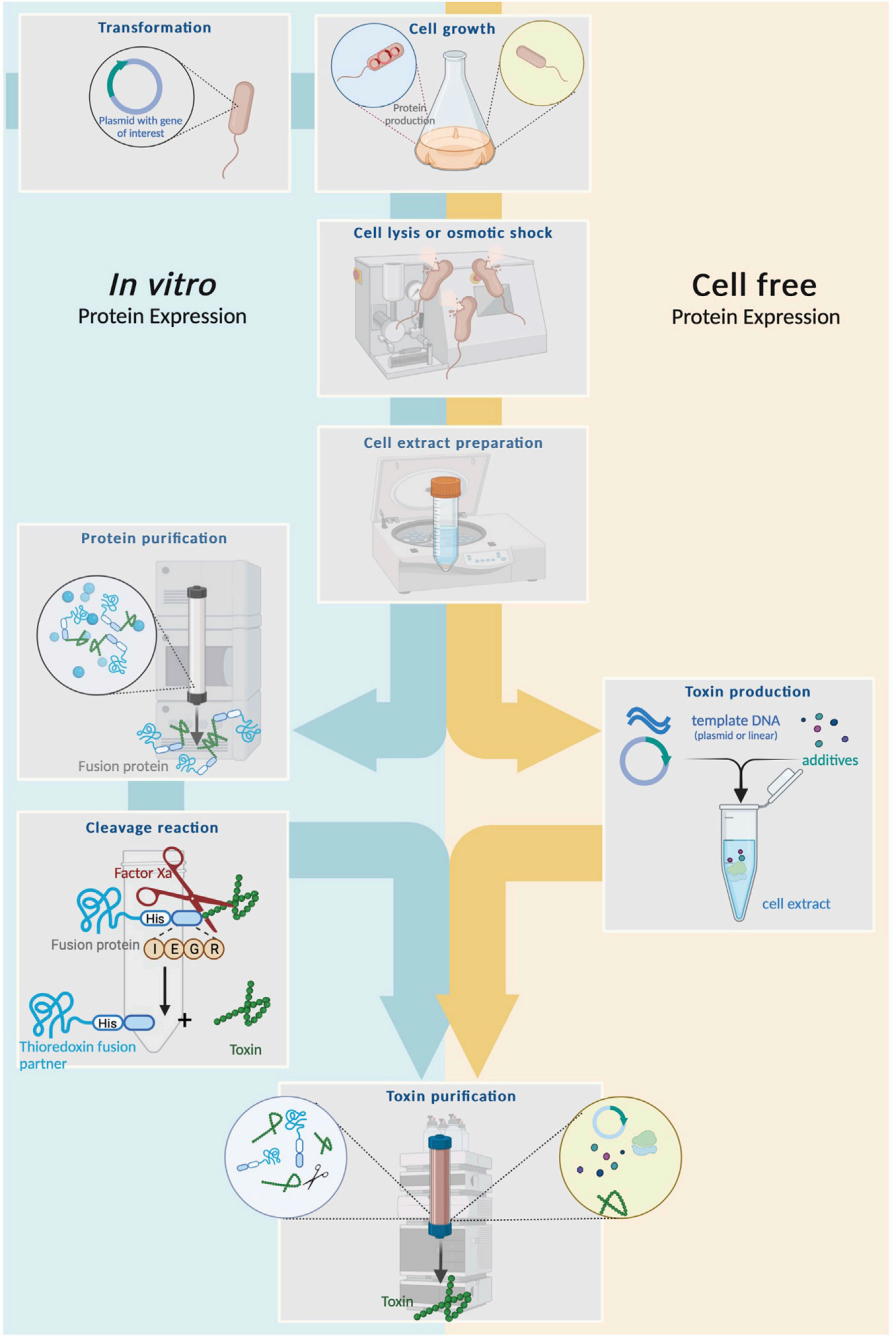
Schematic comparison between heterologous expression and cell-free system for venom toxin production. Left: Heterologous expression begins with cloning of plasmids carrying a gene for the toxin of interest and a fusion partner into a suitable host cell followed by cultivation, lysis and purification of the fusion protein. The latter is subsequentially cleaved with (e.g., by factor Xa) to release the mature toxin. Finally, the toxin is purified via chromatography. Right: Cell-free systems usually start from intact host cells, which are cultivated and lysed to create a cell extract, serving as the primary expression medium. Template DNA encoding the toxin of interest (either a linear gene fragment or a plasmid) plus additives are added and protein synthesis is facilited by the cell lysate. From here, produced toxin can be isolated from the extracted via chromatography.

The production of soluble and correctly-folded venom peptides can be improved by adjusting certain process parameters, such as lowering the production temperature (Semadiras et al., 2013; Chassagnon et al., 2017) or optimizing the medium composition (Nielson et al., 2019; Bertelsen et al., 2021). Although complex media such as lysogeny broth (LB) are sufficient for most small-scale cultures, rich media such as terrific broth (TB) containing glycerol as the carbon source promote higher cell densities and protein expression levels (Volontè et al., 2010; Tripathi, 2016). The simultaneous optimization of multiple process parameters can be achieved by statistical experimental designs based on response surface methodology (Tripathi, 2016). In addition to the inefficient formation of disulfide bonds, bacteria are unable to carry out most of the typical post-translational modifications that occur in eukaryotic cells, such as glycosylation and serine/threonine phosphorylation (Huang et al., 2012; Dalton and Barton, 2014; Wells and Robinson, 2016). This is a major drawback in venom research because many toxin groups feature such modifications, which strongly influence their bioactivity (Rivera-de-Torre et al., 2022). Accordingly, eukaryotic cell lines are promising host systems for toxin production and may complement the use of prokaryotic expression systems. However, eukaryotic systems have only sporadically been used for venom research, as discussed in detail elsewhere (Rivera-de-Torre et al., 2022). Figure 4 outlines a classical heterologous expression experiment and Table 1 highlights some selected toxins expressed via different approaches.

**TABLE 1:** Selected venom components produced via different methods. Given are selected venom toxins discussed in this text that are produced via synthesis, recombinantly od via cell-free approaches. Further indicated are the species from which the toxins were identified, size, modifications and the respective production system used.

Type	Toxin	Species	Size	Modifications	ProductionSystem	References
Synthesis	ShK-toxin	*Stichodactyla helicanthus* (anemone)	4.1 kDa	Disulfide bonds (3x)	Fmoc	Pennington et al., (1995)
Synthesis	Mar1a	*Machimus arthriticus* (robber fly)	3.1 kDa	Disulfide bonds (3x)	Fmoc	Drukewitz et al., (2018)
Synthesis	Protoxin II	*Thrixopelma pruriens* (spider)	3.8 kDa	Disulfide bonds (3x)	Fmoc	Park et al., (2012)
Synthesis	Checacin-1	*Chelifer cancroide*s (pseudoscorpion)	n.a.	Amidation	n.a.	Krämer et al., (2022)
Synthesis	Salamandrin-I	*Salamandra Salamandra* (amphibian)	1.4 kDa	Amidation	Fmoc	Plácido et al. (2020)
Recombinant	Huwentoxin-IV	*Ornithoctonus huwena* (spider)	4.1 kDa	Disulfide bonds (3x)	SHuffle T7 Express lysY	Sermadiras et al., (2013)
Recombinant	H-Vc7.2	*Conus victoriae* (cone snail)	2.8 kDa	Disulfide bonds (3x)	CyDisCo	Nielson et al., (2019)
Recombinant	Conk-S3	*Conus striatus* (cone snail)	n.a.	Disulfide bonds (2x)	pcsDisCoTune	Bertelsen et al., (2021)
Recombinant	Hi1a	*Hadronyche infensa* (spider)	8.6 kDa	Disulfide bonds (6x)	BL21 (DE3)	Chassagnon et al., (2017)
Recombinant	S64	*Sicarius dolichocephala* (spider)	3.6 kDa	Disulfide bonds (3x)	BL21 (DE3)	Loening et al., (2013)
Recombinant	S67	*Sicarius dolichocephala* (spider)	2.1 kDa	Disulfide bonds (3x)	BL21 (DE3)	Loening et al., (2013)
Cell-free	S67 (USCTX)	*Sicarius dolichocephala* (spider)	2.1 kDa	Disulfide bonds (3x)^a^	NEB PURExpress	Lüddecke et al., (2021)
Cell-free	Preprosecapin	*Apis mellifera* (honeybee)	n.a.	n.a.	Wheat germ-based	Vlasak and Kreil (1984)
Cell-free	Kallikrein	Undet. Snake	n.a.	n.a.	Wheat germ-based	Wang et al. (2012)
Cell-free	Pn3a	*Pamphobeteus nigricolor* (spider)	4.2 kDa	Disulfide bonds (3x)	AC-assisted Ec CFS	Wu et al. (2022)
Cell-free	Dc1a	*Diguetia canities* (spider)	6.5 kDa	Disulfide bonds (4x)	AC-assisted Ec CFS	Wu et al. (2022)
Cell-free	HT-1	*Ixodes holocyclus* (tick)	7.8 kDa	Disulfide bonds (4x)	AC-assisted Ec CFS	Wu et al. (2022)

^a^ Correct folding not shown.

#### 3.2.3 Cell-free approaches as an emerging technology in venom bioprospecting

Cell-free protein synthesis, in which proteins are synthesized in cell lysates, can overcome many of the technical and economic drawbacks of chemical synthesis and recombinant DNA technology. The cell lysates are mixed with a DNA template (linear gene fragment or plasmid) encoding a venom component driven by a suitable promoter, and optionally with additives supporting disulfide bond formation (Carlson et al., 2012; Krinsky et al., 2016; Perez et al., 2016; Lu, 2017; Chiba et al., 2021) (see Figure 4). Cell-free production is rapid and scalable, allowing multiplexed small-scale production for screening and the larger-scale production of the most promising venom components. The earliest report was the production of honeybee preprosecapin in wheat germ lysates without subsequent bioactivity screening (Vlasak and Kreil, 1984). The snake venom kallikrein was later expressed in the same system, and the bioactivity was similar to its natural counterpart *in vitro* (Wang et al., 2012). Other cell-free production systems have recently been compared for their ability to produce an ICK toxin from the sicariid spider *Hexophthalma dolichocephala* (Lüddecke et al., 2021). This showed that only one system (based on *E. coli*) was able to produce a bioactive toxin in low amounts and unclear folding, whereas the other systems failed to yield any product at all (Lüddecke et al., 2021). The authors therefore recommended the optimization of cell-free systems and protocols to maximize protein yield and folding specificity (Lüddecke et al., 2021). The most recent application of cell-free systems for venom toxin production involved the modification of a thermodynamically controlled *E. coli*-based cell-free system (AC-assisted Ec CFS) to produce a variety of complex, bioactive folded peptides from a range of sources. These included the spider toxins DC1a (*Diguetia canities*) and Pn3a (*Pamphobeteus nigricolor*) as well as the tick toxin HT-1 (*Ixodes holocyclus*) (Wu et al., 2022). All these toxins are heavily cysteine-crosslinked, showing that cell-free systems can produce challenging venom components (Wu et al., 2022).

#### 3.2.4 Building up on natural templates: rational design to create artificial toxins

In addition to facilitating the production of natural toxins, synthetic biology can also be used to create novel peptides by modifying, augmenting, or even combining parts of known sequences in order to maximize the potential for translational research or identify functionally important amino acids that may increase therapeutic efficacy. For example, venom AMPs from scorpions of the genus *Urodacus* have been modified to reduce their cytotoxicity while maintaining their antimicrobial activity (Luna-Ramirez et al., 2017b). An artificial peptide from *Mesobuthus martensii* was used to disrupt *Pseudomonas aeruginosa* biofilms during and after formation (Teerapo et al., 2019). Peptides from *Vaejovis mexicanus* were engineered to reduce cytotoxicity but increase anti-tumor and antimicrobial activity, as well as the ability to inhibit unicellular parasites such as *Plasmodium* and *Trypanosoma* spp. (Pedron et al., 2017; Pedron et al., 2018; Pedron et al., 2021). Wasp venom AMPs have been modified to achieve similar aims (Torres et al., 2020). A cone snail peptide from *Conus lividus* that binds to nicotinic acetylcholine receptors (nAChRs) was recently engineered to increase selectivity for nAChR α3β2 by > 2000-fold (Zhu et al., 2020). Although most natural venom peptides show potent bioactivity, they are susceptible to proteolytic digestion, which reduces their pharmacological half-life (Peigneur et al., 2021). This has been addressed by developing cyclic analogs of spider and cone snail venom peptides (Peigneur et al., 2019a; Giribaldi et al., 2020; Li et al., 2020; Peigneur et al., 2021).

One of the most innovative approaches to design artificial toxins is the creation of chimeric molecules that combine the activities of two or more parental peptides. For example, heterobivalent ligands of sodium channels consisting of *µ*-conotoxin KIIIA and a derivative of huwentoxin-IV were recently prepared using a range of polyethylene glycol linkers (Peschel et al., 2020). The *µ*-conotoxin KIIIA blocks sodium channel pores whereas huwentoxin-IV is an allosteric gating modulator, so the heterobivalent peptide can interact with two sodium channel binding sites simultaneously, increasing its potency by up to 24-fold (Peschel et al., 2020). Similarly, when cytotoxic phospholipase D from a recluse spider (*Loxosceles gaucho*) was fused to a hemotoxic disintegrin from a viper (*Echis carinatus*), the resulting a chimeric peptide showed synergistic cytotoxic effects against melanoma cells (Siqueira et al., 2019). Venom peptides can also be fused to non-venom components to modify their pharmacological properties. For example, a chimeric ω-conotoxin MVIIA was conjugated to a trans-activator of transcription (TAT) domain, allowing the chimeric peptide to cross the blood–brain barrier and thus overcome a major obstacle for conotoxin-based therapeutics (Yu et al., 2019). Likewise, the anti-diabetic drug exenatide (a toxin from *Heloderma suspectum* venom) was linked to elastin to improve stability (Jung et al., 2019). Finally, fusion proteins based on *Leiurus quinquestriatus* chlorotoxin facilitated drug delivery to glioblastomas (Kasai et al., 2012; Costa et al., 2013; Cohen-Inbar and Zaaroor, 2016; Cohen et al., 2018; Wang et al., 2020b).

#### 3.2.5 Using small-scale biofractionation to isolate low-yield venom components

The production of synthetic or recombinant peptides circumvents the inability to investigate venoms from inaccessible sources. However, the direct analysis of such venom components can still be hampered by low production yields. For example, commercial cell-free systems were unable to deliver high yields of an ICK toxin. In such cases, nanofractionation and picofractionation are promising emerging technologies involving nanoscale liquid chromatography (nanoLC) and the decomplexation and/or fractionation of miniscule sample quantities at the microliter to nanoliter scale (Zietek et al., 2018; Zietek et al., 2019; Slagboom et al., 2020; Still et al., 2020; Xie et al., 2020). This facilitates the biodiscovery of toxins in the venoms of small animals that cannot be milked, as well as the analysis of small amounts of toxins produced by cell-free systems and organoids. The pairing of such fractionation systems with organoid cell culture or other production platforms can therefore help to expand the scope of venom biodiscovery approaches to include almost all venomous animals.

## 4 Methods to study the function of venoms

Synthetic biology can be used not only to produce and study venom components, but also to investigate their mechanisms of action in target species by dissecting the corresponding signaling and response pathways. For example, the creation of synthetic receptor molecules for neurotoxins allows their characterization in electrophysiology experiments (e.g., Peigneur et al., 2019b; Clémençon et al., 2020). More recent biosensor techniques have expanded the scope of mechanistic studies to encompass even more target molecules, simultaneously providing the means to identify specific modes of action, disentangle the phenotypic consequences of venom variation, and infer the mechanistic basis of venom resistance.

### 4.1 Electrophysiology in *Xenopus* oocytes

The ability of neurotoxic venom components to interfere with receptors, such as ion channels, is one of the most widely studied toxin bioactivities. In particular, venoms from snakes, spiders, scorpions and cone snails have been investigated either to understand their potentially life-threatening modes of action, or to probe their interactions with receptors involved in diseases in an attempt to discover new therapeutics (Little et al., 1998; Pawlak et al., 2009; Chassagnon et al., 2017). One widely-used method in this context is the electrophysiological analysis of oocytes from the African clawed frog *Xenopus laevis*. Although this method is widely used, it is often overlooked as an application of synthetic biology. In such experiments, RNA or cDNA encoding a receptor of interest is injected into the oocyte, which is a large, robust cell that is amenable to laboratory manipulation (de Robertis et al., 1977; Ureta et al., 2001; Lin-Moshier and Marchant, 2013). When the receptor is expressed, its function can be determined by patch-clamp analysis. *Xenopus* oocytes can be collected in large numbers and can express a wide range of eukaryotic proteins including membrane-anchored and membrane-spanning receptors (Lin-Moshier and Marchant, 2013). Electrophysiological analysis in *Xenopus* oocytes has been used to study many venoms and toxins, particularly to determine the mechanism of neurotoxic components and to infer the mode of action of potential venom-derived drugs (Takacs et al., 2001; Takacs et al., 2004; Buczek et al., 2007; Peigneur et al., 2019b; van Hout et al., 2019; Clémençon et al., 2020).

### 4.2 Biosensors

A biosensor is an analytical device that combines an immobilized biological element (bioreceptor) recognizing a specific biomolecule of interest with a physical element (biotransducer) that generates a measurable signal (Hall and Hummel, 1995; Pandey and Malhotra, 2019). The bioreceptor can be an enzyme, antibody, nucleic acid or cell, among others (Hall and Hummel, 1995). Biotransducers are classified according to the signal they generate, such as electrochemical, optical, piezoelectric, thermal, or acoustic, and this is also used to define the biosensor as a whole (Pandey and Malhotra, 2019; de Freitas Borges et al., 2021). The first biosensors measured glucose levels in blood by reacting glucose with an oxidoreductase to generate an electrical signal via an oxygen electrode (Clark and Lyons, 1962). The success of biosensors reflects their low cost, long operational life, ease of use, and high sensitivity in a portable, miniaturized device (de Freitas Borges et al., 2021).

In venom research, biosensors can be used for the diagnosis of envenomated patients by identifying venom markers unique to different sympatric species. This is most commonly applied to diagnose snakebites, which are treated using polyvalent antivenoms matching multiple local species if possible, but in many cases only monovalent antivenoms are available and therefore an accurate diagnosis is required. Several biosensors are being developed to identify venomous species by testing blood samples from envenomated patients. Two recent studies describe immunosensors to detect the venoms of *Bothrops* pitvipers and Indian cobras (*Naja naja*), the clinically most relevant snake species in Brazil and India, respectively (de Faria et al., 2018; Choudhury et al., 2018). The bioreceptor in both devices is a specific antibody that recognizes a unique venom component, whereas the transducer in one case is an iron/chrome-coated stainless-steel electrode and in the other is a silver-coated glass slide. Venom binding to the antibody-coated transducer causes a faradaic protein layer to reduce the electric current in the electrochemical biosensor or to alter the angle of light detected by surface plasmon resonance (SPR) spectroscopy, thus returning a positive signal (de Faria et al., 2018; Choudhury et al., 2018).

Biosensors are also used in the search for novel drug leads, such as screening scorpion and snake venoms for AMPs and modulators of nAChRs (Bao et al., 2019; Zdenek et al., 2019), screening snake venom disintegrins for the ability to destroy brain glioblastomas (Er et al., 2019) and searching for antibodies against hemorrhagic snake venom metalloproteinases (Schneider et al., 2014). In the future, biosensors may also be used to identify venoms that target specific pest insects and can therefore be deployed as biopesticides (Windley et al., 2012).

#### 4.2.1 Next-generation biosensors shed light on venom plasticity and resistance

Biolayer interferometry (BLI) is a label-free, microfluidics-free optical technique that allows real-time precision measurements based on the attachment of ligands, which progressively adhere and accumulate, to the surface of an optical fiber-coated biosensor (Apiyo, A., 2022). Once the ligands are attached to the biosensor, they are exposed to the analyte molecules to detect analyte–ligand binding. The biochemical analyte–ligand interactions cause a quantifiable shift in the wavelength of reflected light, which is detected via the fiber optic biosensor. This also provides quantitative data on binding kinetics, such as k_a_, k_d_ and K_D_ (k_a_/k_d_) values. BLI is particularly useful for the biodiscovery and testing of drug candidates and the assessment of antibody quality.

The Octet BLI system (Sartorius, formerly ForteBio) uses short synthetic peptides (13–14 amino acids) known as mimotopes based on loop-C of the orthosteric site of nAChRs to determine the binding interactions of venom *α*-neurotoxins, particularly the 3FTx proteins that generally target this domain (Zdenek et al., 2019). This is based on earlier work in which short nAChR sequences were used to identify the specific binding region and binding mechanics of α-bungarotoxin and other snake venom 3FTx proteins (Bracci et al., 2001; Kasher et al., 2001; Bracci et al., 2002; Katchalski-Katzir et al., 2003). More recently, the mollusk acetylcholine-binding protein and chimeric forms of human *α*-7 orthosteric site residues have been used to investigate the potency of snake venoms and their potential therapeutic use as decoy proteins (Slagboom et al., 2018; Albulescu et al., 2019). However, these studies did not use BLI, instead opting for SPR spectroscopy, a microfluidics system with drawbacks such as clogging, expensive gold sensor chips, and low throughput data retrieval.

Importantly, synthetic nAChR mimotopes have not been used to investigate the evolution and ecology of snake venom toxin–target interactions. BLI was designed to address such evolutionary questions, as well as the potential for biodiscovery (Zdenek et al., 2019). In contrast, the α-1 nAChR (the only nAChR located at the postsynaptic neuromuscular junction, controlling muscle contraction) has been used for evolutionary investigations. These receptors are targeted by α-neurotoxins, which prevents acetylcholine binding and immobilized prey by flaccid paralysis. One drawback of this method is that it only tests binding at the loop-C orthosteric site, and thus cannot detect toxins that bind to nAChRs allosterically or at other positions. However, assuming no complex whole-receptor conformational shape changes are needed for toxin–receptor binding, it should be possible to test any amino acid sequence beyond the orthosteric site for binding to venom toxins.

It must be noted that these studies did not assess any binding kinetics (K_a_, K_d_ or KD) since, firstly, whole venoms were used, and binding kinetics data/affinity constants cannot be determined without a known concentration of the binding analyte. Secondly, these studies were only trying to determine if specific venoms have the capacity to bind to different mimotopes across a range of natural prey orthosteric sites. Binding kinetics data is possible to determine for single toxins within a venom, and future studies should aim to assess this, particularly for bioprospecting purposes.

BLI based on nAChR mimotopes has been highly successful (Harris et al., 2020a; Harris et al., 2020b; Harris et al., 2020c; Harris et al., 2020d; Youngman et al., 2021), but other ion channel receptor domains have been tested in preliminary experiments, including domain IV Ca_V_1.2 (Harris et al., 2021b) and domain IV Na_V_1.4 (Dobson et al., 2021). Further investigations are needed to confirm the initial results. BLI could benefit greatly from being used in conjunction with whole receptor-based electrophysiology methods, particularly when dealing with complex ion channel interactions.

#### 4.2.2 Prey-specific venom targeting

The evolution of predatory venoms is driven by the need to immobilize prey, and some venom toxins target particular prey species (Fry et al., 2003; Mackessy et al., 2006; Pawlak et al., 2006; Pawlak et al., 2009; Heyborne and Mackessy, 2013; Lüddecke et al., 2022). The investigation of prey-specific venoms requires a high-throughput method that covers broad taxonomic diversity, preferably without the need to inject venom into live animals (a contentious ethical issue). BLI provides an ideal solution, allowing animal-free testing in a high-throughput manner.

The exploration of publicly available α-1 nAChR sequences has revealed many amino acid variations between taxa in the loop-C region, even between closely related species (Khan et al., 2020). BLI has allowed the binding of crude venoms rich in α-neurotoxins to be investigated by the assessment of taxon-specific representative sequences across a range of prey (amphibian, mammalian, and reptilian). Prey-specific targeting of taxon-specific mimotopes has been assessed in snakes (Harris et al., 2020a; Harris et al., 2020b; Harris et al., 2020c; Youngman et al., 2021), lizards (Dobson et al., 2021), and even fish (Harris et al., 2021b). All these studies provided insight into the biochemistry and evolution of venoms. There is a lack of publicly available sequences for certain taxa, but most sequences seem to be ubiquitous across large clades, meaning that representative species can be used to provide the vital information. Some studies have taken a step forward in characterizing more of these sequences across diverse clades (Khan et al., 2020). The availability of more genomic data in the future will provide greater access to diverse taxa and will thus provide more insight into the prey-specificity of venom.

#### 4.2.3 Venom toxin resistance

Given the reciprocal nature of evolutionary selection pressure, the prey-specificity of venom toxins drives antagonistic selection for target adaptation (Thiel et al., 2022). This has resulted in the evolution of resistance elements at the loop-C target site, which have also been discovered using the BLI biosensor method.

Early investigations into resistance elements at α-1 orthosteric sites provided fundamental information about the mechanism of resistance (Barchan et al., 1992; Barchan et al., 1995; Kachalsky et al., 1995; Takacs et al., 2001; Takacs et al., 2004; Drabeck et al., 2015). Most of these studies only provided hypothetical examples of resistance, but the use of BLI to examine resistance across the natural prey of venomous snakes confirmed a hypothesized form of resistance while characterizing a novel form of resistance against snake venom toxins (Harris and Fry, 2021). Similarly, nAChR resistance features against snake venom toxins have also been discovered in varanid lizards (Jones et al., 2021) and some primate groups, revealing a deep co-evolutionary relationship (Harris et al., 2021a). This cross disciplinary approach has therefore bridged the gap between biomolecular interactions and the ecology and evolution of venomous animals and their toxins.

## 5 Perspective

Biotechnology has driven the growth of knowledge in many areas of the life sciences, revolutionizing basic and applied research by providing the tools to dissect and manipulate biological systems, including animal venoms. Here, we discussed these methods and the insights gained at three levels of biological organization. At the genomic level, we found that functional genomics methods, particularly RNAi and CRISPR/Cas9, provide information about the processes underlying the formation of venom systems, offering pathobiochemical insights into the process of envenomation from a victim’s perspective. At the level of gene products (toxins), we discussed the potential of organoids and other cell culture technologies to produce crude venom in the laboratory without animal breeding facilities. We also considered the production of selected venom components by chemical synthesis, heterologous expression in prokaryotic and eukaryotic cells, or cell-free systems. These methods allow the analysis of natural venom components from lineages that are inaccessible via traditional biofractionation due to insufficient venom yields. They can also be used to produce designed venom-inspired proteins with desirable physicochemical properties leading to novel therapeutics, agrochemicals or molecular tools. Moreover, we discussed how synthetic biology and biotechnology can be used at the level of the phenotype to understand the activity of venom and its components, particularly by developing bioassays and biosensors to study the complex functions and exochemistry of venoms as well as their plasticity and evolution with increasing precision and speed.

**FIGURE 5:**
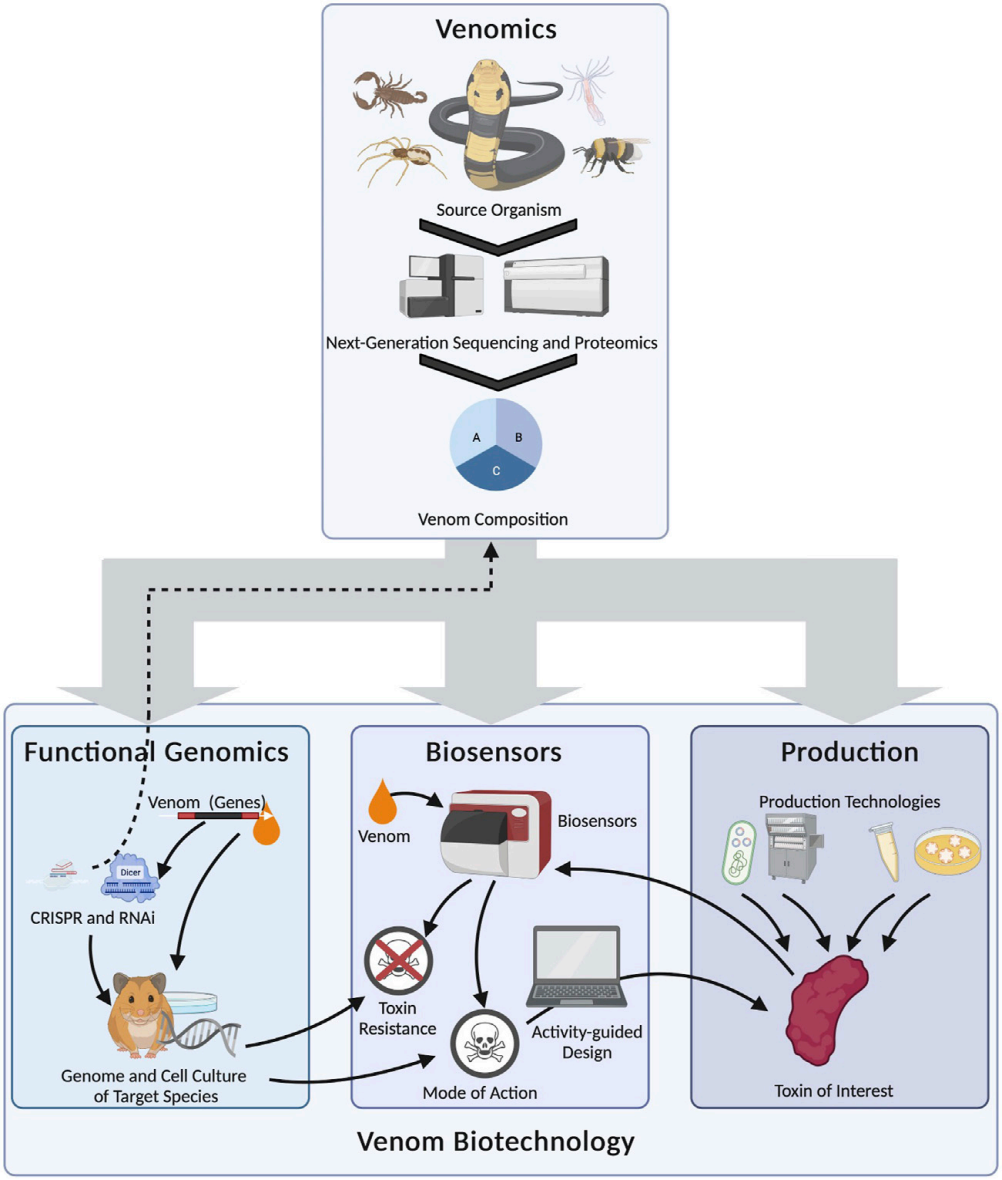
Selected examples of the complex relationship between venom biotechnology and venomics, and the multilateral interconnections between biotechnological fields in venom research (arrows). Venomics serves as the foundation for the subsequent biotechnological analysis of venom systems by providing fundamental information about the venom system. Functional genomics can then be used to discover the genetic basis of complex traits, such as venom regulation or venom susceptibility. It can also be used to modify the original venom systems (dashed arrow). Biosensors can be used to infer venom modes of action and resistance. As well as providing information on the role of venom in natural scenarios, the data can be used to design artificial toxins with desired activities for drug discovery programs. These, and also the natural toxins identified by venomics, can be accessed using different biotechnological production systems (from left to right: heterologous expression in prokaryotes, chemical synthesis, cell-free systems and organoids). The toxin of interest can then be fed back to the biosensor platform to determine its function and any potential translational applications.

The venom biotechnology toolkit provides fundamental insights into venom systems, particularly when different technological approaches are combined (Figure 5). For example, the analysis of high-quality genomes spanning additional venomous taxa will broaden the range of organisms amenable to RNAi and CRISPR/Cas9 screening. Organoids from neglected venomous animals may, when combined with microscale fractionation, be used to isolate venom components from species that have been inaccessible thus far using traditional techniques. Combinations of different expression technologies can also be compared to identify the most appropriate systems for different components, and the high-throughput capabilities of cell-free systems in particular may pave the way toward high-throughput screening for a broad range of bioactivities. The most promising components can then be produced in larger quantities for more detailed testing using advanced assays and biosensors. These examples highlight the power of synthetic biology and biotechnology to unravel some of the most fascinating aspects of venom biology, to understand the activities of venom components, and to repurpose these lethal natural weapons for the benefit of humans.
